# *Alicyclobacillus acidoterrestris* Eradication Strategies with Physical Methods and Natural-Origin Substances Intended for Food Industry

**DOI:** 10.3390/molecules31020257

**Published:** 2026-01-12

**Authors:** Agnieszka Tyfa, Alina Kunicka-Styczyńska

**Affiliations:** 1 Institute of Agricultural and Food Biotechnology—State Research Institute (IBPRS-PIB), Food Quality Department, Al. Marszałka J. Piłsudskiego 84, 92-202 Lodz, Poland; agnieszka.tyfa@ibprs.pl; 2 Department of Sugar Industry and Food Safety Management, Faculty of Biotechnology and Food Sciences, Lodz University of Technology, Wólczańska 171/173, 90-530 Lodz, Poland

**Keywords:** *Alicyclobacillus acidoterrestris*, food industry, biofilms, essential oils, food control, food spoilage

## Abstract

*Alicyclobacillus acidoterrestris* is an acidothermophilic bacterium considered a significant challenge to the food industry, particularly in the production of fruit juices and concentrates. Its ability to survive pasteurization and form spores and biofilms makes it a persistent contaminant that can spoil products and generate off-flavors even during product storage. Recent studies have increasingly focused on developing new strategies to eliminate both vegetative cells and biofilms, with special attention on natural compounds such as plant extracts, essential oils and antimicrobial metabolites. These natural agents offer promising alternatives for controlling *A. acidoterrestris* and might contribute to improvement in safety and quality of juice products. This article presents a comprehensive overview of current strategies for controlling *Alicyclobacillus* species in food processing environments, with an emphasis on *A. acidoterrestris* as a major spoilage organism in the fruit juice industry. It summarizes the established physical and chemical control methods, as well as highlights emerging novel approaches involving natural-origin antimicrobial compounds considered useful for mitigating *Alicyclobacillus* contamination.

## 1. Introduction

Microbiological contamination is one of the food industry’s key problems, causing economic losses and posing a potential threat to consumer health. In recent years, there has been an increase in the spoilage of juices and fruit drinks in the food industry’s juice sector, caused by the growth of acidothermophilic bacteria belonging to the *Alicyclobacillus* genus. The presence of these microorganisms in finished products (fruit juices and concentrates) may indicate contamination at the primary production stage, including the quality of raw materials, pre-production conditioning and transport to the processing plant. If secondary microbiological contamination occurs at any of these stages, *Alicyclobacillus*, due to their specific characteristics (spore formation, acid and heat tolerance) [1], can survive all stages of the production process. Several strains of these bacteria that can spoil juice products have been described, and *Alicyclobacillus acidoterrestris* is the species most commonly isolated [2]. To date, research on these bacteria has mainly focused on the characteristics of *A. acidoterrestris* and methods to prevent and eliminate it from production, taking a wide range of methods into account [3]. This includes physical (both thermal and non-thermal) and chemical methods, as well as those involving plant and microbial metabolites. Few studies have addressed the colonization of abiotic surfaces by strains originating from natural primary production environments, as well as methods for their elimination [4,5,6]. Microbial colonization remains problematic for the food industry due to the formation of biofilms on machinery and technological equipment surfaces. Microorganisms with adhesive properties adhere to surfaces and then form aggregates and microcolonies, which are stabilized by extracellular polymeric substances (EPSs). This process results in the formation of biofilms [7,8]. Furthermore, biofilms are more resistant to environmental factors than planktonic forms. The mature biofilm structure is more difficult to eradicate, which facilitates cell spreading and colonization of other surface areas. This review outlines the properties of *Alicyclobacillus* spp., with a particular focus on *A. acidoterrestris*, and describes methods for controlling and removing these acidothermophilic bacteria and their biofilms from industrial environments.

## 2. *A. acidoterrestris* as a Threat to the Food Industry

*A. acidoterrestris* were first isolated in Germany in 1982 from spoiled pasteurized apple juice [9]. Since then, they have been recognized as acidothermophilic bacteria that are most commonly found in fruit and vegetable products. Food spoilage caused by *A. acidoterrestris* has been reported in various regions of the world, including Europe, Japan and the USA. The bacteria have been isolated from various fruit juices and concentrates, including pineapple, peach, grapefruit, apple, raspberry, coconut milk, orange, tomato, strawberry, cherry, chokeberry, white grape, blackcurrant, mango and passion fruit. They have also been found on the surfaces of raw materials such as pears, apples and kiwis [2,10,11,12,13,14,15,16,17,18,19,20]. Other studies have shown that *A. acidoterrestris* can also grow in lemon juice, emulsions used in juice production and beverage additives [21,22,23]. Contamination of food products by *A. acidoterrestris* is difficult to detect. These bacteria do not produce gases; therefore, no gassing of juices or ‘bloating’ of packaging is observed. Product spoilage may be accompanied by cloudiness or the formation of a white sediment in cases of high bacterial growth [24,25,26]. The most common and easily detectable symptom of juice spoilage by *A. acidoterrestris* is a change in the product’s organoleptic characteristics, caused by the bacteria producing odorous compounds. Depending on the temperature, a slight decrease in pH may also occur [27]. Strains of *A. acidoterrestris* have been proven capable of producing the volatile compounds 2-methoxyphenol (guaiacol) and halophenols, including 2,6-dibromophenol (2,6-DBF) and 2,6-dichlorophenol (2,6-DCF), with the amount of guaiacol exceeding the concentration of other aromatic compounds by about 1000 times [11,12,24,26,28,29,30].

Guaiacol has a distinctive smell that is often described as “sweet”, “smoky”, “phenolic” or “medicinal”. Its precursors include ferulic acid and vanillin. Ferulic acid is a key component of lignin, a substance commonly found in plant cell walls. With the help of bacterial enzymes, it is metabolized into vanillin, vanillic acid and protocatechuic acid, which can then be converted into guaiacol. Another guaiacol precursor is tyrosine occurring naturally in apple juice [24]. It is believed that the amount of guaiacol produced from tyrosine depends on the heat shock applied in the production process and the juice storage conditions [31]. Due to its intense smell and taste, and its low sensory sensitivity threshold, guaiacol is easily detectable. The olfactory detection threshold for guaiacol is 0.021 ppm in water, 0.07 ppm in oil and 0.03 ppm in 12% ethanol. The taste sensitivity threshold concentration of guaiacol in water is 0.013 ppm [26]. Various sources state that the olfactory detection threshold for guaiacol in apple juice, orange juice and non-carbonated fruit drinks is 0.48, 0.91 and 0.57 ppb, respectively [32,33]. The taste threshold for guaiacol in these products is 0.24 or approximately 2 ppb [32,34], while the recognition threshold is 2 or 2.23 ppb [33,35]. The level of guaiacol produced is related to the degree of *A. acidoterrestris* growth. Depending on the test method, guaiacol was detected in apple, grapefruit and orange juices at a bacterial cell count of 10^4^–10^5^ CFU/mL [12,36]. In juices stored at room temperature, where growth of *A. acidoterrestris* is limited, the guaiacol level was found to be significantly lower than in juices incubated at 37–46 °C [12,26,35].

Other major metabolites of *A. acidoterrestris* include the halogenated hydrocarbons 2,6-dibromophenol (2,6-DBF) and 2,6-dichlorophenol (2,6-DCF), with much lower sensory detection levels. The olfactory detection threshold in water is 0.5 ng/L for 2,6 DBF and 6.2 ng/L for 2,6 DCF, and the smell is described as ‘disinfectant’ or ‘hospital-like’ [28,37]. In fruit juices, the concentrations of these two compounds reach 2–4 ng/L and 16–20 ng/L, respectively. The recognition threshold for 2,6-dichlorophenol is 20 ng/L [38]. In addition to 2,6-DBF and 2,6-DCF, some strains of *A. acidoterrestris* can produce other metabolites in pineapple, apple and mango juices that smell “cheesy” and “sulfurous”: 2-methylbutyric acid, 3-methylbutyric acid, and 3-(methylthio) methylpropionate [16].

## 3. Removal and Control of *A. acidoterrestris* from Industrial Environments and Food Products

*Alicyclobacillus* spp., as acidophilic and thermophilic microorganisms producing heat-resistant spores, are a common and difficult-to-eliminate source of microbial contamination in fruit processing environments [26,31]. Although viable cells of *A. acidoterrestris* are eliminated by applying standard pasteurization conditions, the spores survive and are activated by heat shock, resulting in bacterial growth in finished, cooled and stored products. Therefore, typical pasteurization parameters do not ensure that food products maintain adequate quality and microbiological purity. The alternative solution is to increase the temperature to sterilization or UHT (ultra-high-temperature processing) parameters. However, this usually causes undesirable changes in the taste and color of fruit juices. Until now, ongoing research has provided knowledge on the behavior of *A. acidoterrestris* in different model environments, including the prevention of cell growth and spore germination in various conditions [39,40,41], used to search for technological and methodological solutions to control and eliminate both *A. acidoterrestris* cells and their biofilms. The alternative solution, however, cannot cause unfavorable organoleptic changes in food products or negatively affect their quality.

### 3.1. Physical Methods

According to Lee et al. [42] and Alpas et al. [43], the simultaneous use of pasteurization and elevated pressure (HPP, high-pressure pasteurization; HHP, High Hydrostatic Pressure) results in the inactivation of *A. acidoterrestris* spores. The number of spores in apple juice may be reduced below the detection level after 10 min of exposure to pressures of 414 and 621 MPa at 71 °C [42,43]. A similar level of spore reduction was obtained with a shorter exposure time at 90 °C [42]. For orange, apple and tomato juices, the number of spores decreased by approximately 4 log CFU/mL after a 10 min exposure to 350 MPa at 50 °C [43]. High-pressure pasteurization carried out in buffers and tomato sauce under model conditions was effective at pH in the range between 4.0 and 4.2 [44]. Additionally, it was found that, at lower temperatures, pasteurization was more effective at lower pressures and, at higher temperatures, at higher pressures. As demonstrated by Skąpska et al. [45], HPP of apple juice at 50 °C using pressures of 300 and 500 MPa is equally effective. The lower pressure was, however, characterized by greater effectiveness. The most satisfactory results were obtained using a two-phase method: 30 min of exposure to a constant elevated pressure of 500 MPa at 50 °C, preceded by pulsed pressure (6 × 100 MPa at 50 °C for 5 min) and followed by one hour of incubation at 50 °C [45]. To inactivate *A. acidoterrestris* in malt extract broth, Bevilacqua et al. [46] used high-pressure homogenization (HPH). They found vegetative cells to be more susceptible than spores, probably due to wall protein disruption [2].

Non-thermal methods of removing and controlling the presence of *A. acidoterrestris* in food include radiation (infrared and ultraviolet), microwaves, ultrasound, ultrafiltration and high CO_2_ pressure. Bahceçi et al. [12] demonstrated that ultrafiltration may be an ineffective method of preventing and eliminating acidophilic thermophilic bacteria due to spores passing through membrane filters. Infrared radiation (IR) is regarded as an effective method of combating spore-forming microorganisms in low-moisture food products [26]. IR radiation causes direct changes at the DNA level, contributing to cell death. In studies by Nakauma et al. [47], *A. acidoterrestris* were subjected to gamma radiation in combination with traditional pasteurization, reducing spore inactivation time to 23 min. Similarly, significant reduction in alicyclobacilli spore count was observed in apple and orange juice concentrates [48]. On the other hand, some confirm that ultraviolet radiation (UV) is equally effective, as only a 15 min exposure to UV light reduced the number of *A. acidoterrestris* spores in apple and white grape juice by 2.0–5.5 log CFU/mL [49]. Moreover, high efficiency of combined methods increases the lethality of the bacteria in apple juice [50].

High-amplitude sound waves, or ultrasound, are used in the food industry mainly to disperse and remove bacterial biofilms [51]. However, a study by Yuan et al. [52] on *A. acidoterrestris* in apple juice found that a 30 min exposure to ultrasounds (23 kHz) was sufficient to inactivate over 50% of the bacterial load. The longer time applied resulted in greater inactivation efficiency [52]. Similar effects were obtained using the integrated ultrasound and pulsed electric field (US-PEF) method [53,54]. Unfortunately, a disadvantage of both methods is the alteration of the organoleptic and physicochemical properties of fruit juices.

Microwaves are considered a rapid and easy method of controlling microbial growth. Unlike traditional thermal methods, it has been proven that microwaves cause fewer organoleptic changes in food products [55]. This method’s effectiveness against *A. acidoterrestris* was shown by a twofold reduction in the number of spores in asparagus cream at various selected power and time settings [56]. Studies on the inactivation of spores in apple juice have also been conducted, showing that effective microwave action requires 720 W for 40 s [57].

One of the unconventional methods of combating *Alicyclobacillus* is the use of active antimicrobial packaging, where the packaging may contain active biological or chemical agents, or metal ions, such as silver, copper or platinum [24,58]. In the USA, the U.S. Food and Drug Administration (FDA) currently permits the use of AgION technology, which involves the diffusion of silver ions directly into food, which has also been confirmed to be effective against vegetative cells of *A. acidoterrestris*, preventing their multiplication [59].

Washing the surface of fruit with a chlorine dioxide solution and gaseous ClO_2_ usage has also been recommended; however, this method has limited application due to the deterioration of fruit quality [24,60,61].

An alternative method is the use of GRAS (Generally Recognized as Safe) gases such as high-pressure carbon dioxide (HPCD) or ozone [26]. Studies by Bae et al. [62] presented a reduction in the number of *A. acidoterrestris* spores below the detection level in apple juice to which carbon dioxide was introduced at pressures of 100 or 80 bar for 40 or 30 min, respectively, at temperatures of 65 or 70 °C. The authors did not observe any organoleptic changes in the apple juice [62]. Following ozonation of the apple juice, the reduction in spore count ranged from 1.8 to 2.8 log CFU/mL depending on the ozone dosage and process temperature [63].

The application of physical methods to eliminate alicyclobacilli in industrial practice usually involves introducing additional technological operations, while modifying technological production lines or lengthening the production process generates higher production costs. Using physical factors to inhibit spore germination and limit the growth of A. *acidoterrestris* creates a risk of organoleptic changes in products, determining the extent and duration of the physical parameters used.

### 3.2. Natural Compounds

Apart from the traditional methods used against *A. acidoterrestris*, substances found in nature that limit the growth of these microorganisms are also being applied. A proven antimicrobial effect has been confirmed in natural compounds of animal, microbial and plant origin, and these properties are often enhanced when combined with thermal processing.

#### 3.2.1. Compounds of Animal Origin

Natural active compounds of animal origin mainly include lysozyme and chitosan. Lysozyme is found in tissue fluids, saliva, serum, granulocytes, macrophages and eggs [64]. It breaks down peptidoglycans in bacterial cell walls by hydrolyzing β-1,4-glycosidic bonds between *N*-acetylglucosamine and *N*-acetylmuramic acid. Lysozyme exhibits antibacterial activity against Gram-positive bacteria, while Gram-negative bacteria are more resistant. Thanks to its GRAS status, granted in 1998, lysozyme is currently widely used in the food industry as a biopreservative for various food types [64]. Lysozyme’s antibacterial activity has also been proven against *A. acidoterrestris*, although the mechanism of action is not yet fully understood. According to Bevilacqua et al. [65], this enzyme induces spore germination and exhibits lytic activity against vegetative cells and germinating spores. The MIC (minimum inhibitory concentration) values determined for *A. acidoterrestris* were 0.1–6.0 ppm for vegetative cells and 0.1–3.0 ppm for spores. Studies were also conducted to determine the antibacterial properties of lysozyme when combined with polymeric matrices. In a primary study by Bevilacqua et al. [66], lysozyme was found to be active toward spores and vegetative cells of *A. acidoterrestris* in acidified malt extract broth and apple juice when applied as a solution or as an actively releasing component of water-soluble polyvinyl alcohol (PVOH) film [66]. Similarly, the suppression of bacterial growth and spore germination was noted by Conte et al. [67] for presumable active antibacterial packaging material, considering lysozyme bonded to a polyvinyl alcohol matrix in both in vitro tests and apple juice. Interestingly, research by Conte et al. [67], Bevilacqua et al. [65] and Sokołowska et al. [68] found that spores were more sensitive to lysozyme than vegetative cells.

Another naturally occurring, animal-derived substance with proven antimicrobial properties is chitosan. This non-toxic polysaccharide is produced by deacetylating chitin, which is found in the shells of crustaceans. Thanks to its broad antimicrobial spectrum, chitosan has a variety of applications in medicine and the food industry. Numerous reports have confirmed its extracellular and/or intracellular effects against bacteria and fungi [69]. However, researchers claim that the precise mechanism of action is determined by several factors, including environmental conditions, the type of microorganism targeted, the molecular weight and the degree of acetylation [70]. High-molecular-weight chitosan has a restricted ability to penetrate cell walls and membranes; therefore, it is believed to act as a metal-chelating agent. On the contrary, low-molecular-weight chitosan not only exhibits extracellular activity but also affects RNA, synthesis of proteins and mitochondrial function [70]. Although scientific knowledge of the impact of chitosan on *A. acidoterrestris* is still limited, research data that has already been published seems promising. The results of a preliminary study by Falcone et al. [71] showed that a 1.4 g/L concentration of low-molecular-weight chitosans inhibits spore germination, especially when combined with heat treatment. According to Ulfadillah and Chang [72], the antimicrobial effect against *A. acidoterrestris* was dose-dependent, and increased with a greater molecular weight of chitosan. Thus, chitosan with a molecular weight of 85 and 164 kDa at concentrations of 75 and 100 ppm was sufficient to provide a bactericidal effect after less than 5 or 10 h of incubation, respectively [72]. Furthermore, it has been stated that chitosan affects cell morphology by disrupting its integrity, which is visible in the formation of perforations in the cell wall and leakage of the cellular matrix. Additionally, it inhibits the development and growth of the spores [72].

#### 3.2.2. Microbial-Origin Compounds and Microorganisms

Bacteriocins are ribosomally synthesized protein metabolites with inhibitory or lytic properties against microorganisms. Most of them are produced by Gram-positive bacteria, but the occurrence of bacteriocins from Gram-negative bacteria, such as *Escherichia coli*, has been confirmed by several research studies [73]. So far, the most extensively studied bacteriocins are those produced by lactic acid bacteria (LAB), and some of them have gained interest in terms of their potential application in the food industry. While many bacteriocins appear to be promising preservative agents that enhance food stability or shelf life, the U.S. FDA has only approved nisin, pediocin and Micocin^®^ as food additives [74].

Nisin is currently the only bacteriocin with both proven antimicrobial activity and GRAS status [75]. Due to its stability at high temperatures and in acidic environments, nisin can be added to fruit juices and pasteurized beverages to inhibit the growth of microorganisms including *A. acidoterrestris*. These properties are highly important for controlling bacterial growth and suppressing spore germination [76]. The efficacy of nisin’s antibacterial activity varies depending on the concentration of the bacteriocin applied, the chemical composition of fruit juices, the water activity and the strain of *A. acidoterrestris* [75,77,78,79]. Splittstoesser et al. [79] observed that, as total extract levels increased, the nisin concentration required to inhibit bacterial growth decreased. Some studies have demonstrated that adding nisin at a concentration of 100 IU/mL to grapefruit, apple and orange juices combined with their incubation at approximately 45 °C effectively inhibited the growth of *A. acidoterrestris* [36,77,80,81,82]. In the course of the research undertaken by Jiangbo et al. [83], it was observed that some *Alicyclobacillus* strains exhibited vigorous growth in kiwi juice when a concentration of nisin below 5 IU/mL was administered. However, no bacterial growth was detected when the nisin concentration was increased to 20 IU/mL [83]. Higher antibacterial activity of nisin was reported in environments with a low pH, as well as greater sensitivity of spores compared to vegetative cells [76]. After 12 days of incubation, no germination of *A. acidoterrestris* spores was observed in orange juice or multi-fruit juice supplemented with 25 IU/mL or 50 IU/mL of nisin, respectively. However, nisin added to apple juice at a concentration of 600 IU/mL showed no bacteriostatic or sporostatic activity. Similarly, the inhibition of spore germination was observed in juices incubated at room temperature at a concentration of 50 IU/mL of this bacteriocin [36]. Furthermore, Khallaf-Allah et al. [84] found that a nisin concentration of 62.5 IU/mL was sufficient to decrease the heat-induced reduction in spores, and a lower concentration could limit spore outgrowth in pasteurized and unpasteurized orange nectar during storage at 25 and 45 °C. Research by Sokołowska et al. [68] suggests that strains of *A. acidoterrestris* isolated from concentrated apple juice, a beverage emulsion, concentrated orange juice, and orange juice were sensitive to nisin. The lowest concentrations at which growth inhibition was observed were 50–1250 IU/mL for vegetative cells and 100–1500 IU/mL for spores, depending on the strain [68]. On the contrary, Molva and Baysal [85] found that adding nisin at a much lower concentration (10 IU/mL) to apple juice inhibited the growth of *A. acidoterrestris* at temperatures between 25 and 43 °C. Apart from the direct addition of bacteriocins to beverages or fruit juices, researchers focus on the possibility of using nanoparticles or modifying product packaging. According to Buonocore et al. [58], matrices containing nisin (≥60 IU/mL), when used as an element of active packaging, inhibit the growth of acidothermophilic bacteria and spore germination by releasing the bacteriocin during storage. Recent studies suggest that functional iron oxide nanoparticles conjugated with nisin could retain antimicrobial properties in a wide pH range [86]. This is of high importance in terms of controlling *A. acidoterrestris* and could be a useful future tool for preventing spoilage and extending products’ shelf life [86]. Nevertheless, the efficacy of nisin could be improved by combining it with other potent or proven inhibitory factors against *A. acidoterrestris*. This could involve adding plant active compounds, modifying the temperature, and applying UV light or high pressure to beverage products [39,87,88].

Amongst the various bacteriocins examined, paracin C displays characteristics analogous to nisin, i.e., efficacy in low-pH environments [89]. Model studies using vegetative cells of *A. acidoterrestris* found that paracin C was bacteriostatic at a concentration of 80 AU/mL and bactericidal at 160 AU/mL. No spore germination was observed following the application of the bacteriocin at a concentration of 400 AU/mL [89]. Further studies have shown that paracin C inhibits the growth of vegetative bacterial cells at a concentration of 30 µg/mL in apple juice, causing significant morphological changes including cell wall damage and cytoplasmic leakage after 24 h of incubation. Endospores remained unaffected, even at concentrations of 200 µg/mL. However, such a high concentration of paracin C decreased the quality of apple juice, as an unpleasant aftertaste was detected [90]. Interestingly, a content of 100 μg/mL of this bacteriocin seemed to weaken the heat resistance of *A. acidoterrestris* spores [90].

Similar properties were observed for spores treated at temperatures ranging from 80 to 95 °C in mango pulp containing bovicin HC5. At different pH values (4.5–7.0), bovicin HC5 inhibited the growth of *A. acidoterrestris* vegetative cells at a concentration of 100 AU/mL. Meanwhile, no spore germination was observed after 36 h of incubation in the presence of bovicin at a concentration of 80 AU/mL [91]. Additionally, the bacteriocin appeared to affect vegetative cells to a similar extent to nisin in acidic tropical fruit juices, with the amount of viable cells being reduced below the detection limit at a concentration of 60–80 AU/mL [92]. The biological activity produced by *Bifidobacterium bifidum* (Bificin C6165) as a bacteriocin against various *Alicyclobacillus* strains has been confirmed under highly acidic conditions (pH 3.5) [93,94]. A bacteriostatic effect was observed when bificin was added at a concentration of 32–64 AU/mL, and the number of viable cells fell below the detection limit at a concentration of 512 AU/mL [94]. Adding bificin C6165 (80 μg/mL) to commercial apple juice [93], as in a study concerning nisin [76], did not affect the growth or germination of *A. acidoterrestris* spores. Simultaneously, bificin at a concentration of 40 μg/mL inactivated *A. acidoterrestris* vegetative cells [93]. On the contrary, the bacteriocin RC 20,975 appeared to be effective against both the vegetative cells and the spores of *A. acidoterrestris.* The bacteriostatic effect in AAM (*A. acidoterrestris* medium) was observed at a concentration of 64 AU/mL, as was the bactericidal effect when the concentration of bacteriocin was doubled. However, the viable spore count was reduced below the detection limit when 256 AU/mL of bacteriocin was added [95].

Other bacteriocins with proven activity could act as natural preservatives in fruit juices by limiting the growth of *Alicyclobacillus* spp. [96,97]. Grande et al. [96] found that enterocin AS 48, at a concentration of 2.5 μg/mL, inhibited the growth of *A. acidoterrestris* in natural apple and orange juices stored at 37 °C. The bacteriostatic effect of these juices persisted for 14 days. For commercial fruit juices, this effect lasted 60–90 days depending on the juice type and storage temperature (4–37 °C) [96]. Minamikawa et al. [97] confirmed the strong antibacterial properties of warnericin RB4 against *A. acidoterrestris* and *Alicyclobacillus acidocaldarius*. It was demonstrated that the bacteriocin retained its activity over a wide pH range (2.0–10.0) and under heat treatment (100 °C, 15 min). In the most current research, the authors claim that plantaricin YKX, a bacteriocin from *Lactobacillus plantarum*, not only inhibits the growth of acidothermophilic bacteria, but also reduces the production of guaiacol [98].

Although the scientific evidence on the effects of various bacteriocins is well documented, much more research concerning their safety and application in food products is needed. Particular attention should be given to substances that are already permitted as active food additives, like pediocin and Micocin^®^, even though they are intended for different types of food.

A recent study by Wang et al. [99] showed that *A. acidoterrestris* growth may also be controlled using natural glycolipids extracted from *Dacryopinax spathularia* fungus. Natural glycolipid inhibited the growth of bacteria at 8 ppm concentration, while higher concentrations (10, 50, 100 ppm) effectively prevented the outgrowth of alicyclobacilli in apple juice stored for four weeks at 45 °C; however, its effect was masked at 25 °C [99]. Despite the promising antimicrobial activity, data concerning the glycolipid influence on *A. acidoterrestris* are still limited.

Novel application methods focus on eliminating bacteria from the food industry using bacteriophages. This alternative is particularly interesting given that phages remain strictly virulent toward a specific target microorganism [100]. In the food industry, phage cocktails could be used as biopreservatives to extend products’ shelf life or as biopreparations designed to prevent microbial biofilm formation at various stages of production or primary production. A recent study by Shymialevich [101] reported on the action of the lytic *Alicyclobacillus* phage strain KKP 3916 against 53 different *Alicyclobacillus* strains. Unfortunately, the phage infected less than 25% of the bacterial strains tested, which implies a rather narrow host range. A broader host range is especially important for the food industry as it enables the control of a greater number of bacterial species, particularly pathogens. Additionally, the impact of environmental conditions such as acidity and temperature on a single bacteriophage should be evaluated to verify its effectiveness and the possibility of applying other methods to eliminate bacteria [5].

#### 3.2.3. Plant-Origin Compounds and Substances

Despite the extensive literature on the antimicrobial activity of essential oils, few publications have examined the impact of essential oils or their components on the growth of *Alicyclobacillus*. The impact of rosemary oil, lemon oil, neroli oil and the components of cinnamon, lemon and clove oils on these bacteria has been analyzed [6,21,39,40,102]. Antimicrobial activity against *A. acidoterrestis* in terms of growth ability and bacterial survival was investigated for the selected natural plant metabolites, such as essential oils and plant extracts (Table 1).

In a study with lemon essential oil, bacterial spore germination was inhibited at concentrations of 0.08%, 0.12% and 0.16%, but the authors reported that the antimicrobial properties of essential oils depend on their chemical composition [21]. The main component of lemon essential oil is generally limonene, the impact of which on *A. acidoterrestris* cells and spores was assessed by Huertas et al. [40] and Bevilacqua et al. [103]. However, the chosen concentrations in both studies (3.7 mM and 100–500 ppm, respectively) were too low to inhibit the growth of acidothermophilic bacteria. The introduction of citral appeared to affect spore inactivation to a greater extent, with a reduction of 1 log CFU/mL observed after the addition of 0.69 mM citral. Furthermore, the mixture of citral and nisin with various ratios showed antibacterial efficacy, reducing spore levels below the detection limit during short-time heating [40]. Numerous studies have shown *A. acidoterrestris*’s susceptibility to plant extracts [104,105,106,107,108,109]. In a study by Bevilacqua et al. [105], the activity of three extracts was compared: lemon, neroli and a citrus extract sold under the brand name Bicitro^®^ (Quinabra, Probena, Spain). After adding each extract at a concentration of 500 ppm, a 5 log CFU/mL reduction in the number of *A. acidoterrestris* spores was observed. However, the neroli extract could not be added to juices at such a concentration due to its strong impact on the taste and smell. It was found that a combination of Bicitro^®^ or lemon extracts (80 ppm) and thermal treatment effectively reduces spore numbers and improves juice stability [105].

Molva and Baysal [107] found that adding grape seed extract to apple juice decreased the viable count of *A. acidoterrestris* by approximately 3.1–4.6 log CFU/mL after 28 days of storage at 37 °C. Interestingly, extracts at concentrations ranging from 0.23 to 3.6% exhibited a bacteriostatic effect on apple juice after 14 days [107]. In another study, the authors observed a 2.8–3.6 log CFU/mL decrease in the number of vegetative cells when introducing 2.5–40 μg/mL of pomegranate fruit extracts to apple juice [106].

Active components of cinnamon and clove oil—cinnamaldehyde and eugenol—express strong antibacterial properties; however, more effective control of the development of *A. acidoterrestris* is observed when these two active substances are combined [6]. A bactericidal effect was obtained with a 20:40 ppm mixture of cinnamaldehyde and eugenol. Furthermore, a mixture of a doubled concentration of these two substances enabled the microbiological stability of apple juice to be maintained at 35 °C for over seven days. However, the authors also noted that, to inhibit the growth of *A. acidoterrestris* in apple juice, active substances, primarily cinnamaldehyde, should be used at lower concentrations than in culture media due to their strong flavoring properties, which contribute to the formation of an unusual aroma [6].

Methanol extracts of 26 *Eucalyptus* species’ leaves were found to have varying effects on alicyclobacilli. Only seven extracts inhibited the growth of *A. acidoterrestris* with MIC 7.8 μg/mL, while four extracts had MICs in the range of 15.6–63.0 mg/L [104]. The addition of rosemary extract to apple juice has been reported to reduce *A. acidoterrestris* spores by 2.0–3.5 log CFU/mL. Furthermore, two rosemary extracts (V20 and V40) at minimum inhibitory concentrations of 7.8 μg/mL and 3.9 μg/mL, respectively, did not cause any organoleptic or physicochemical changes to the apple juice [108]. In the study by Silva et al. [39], a concentration of 125 μg/mL of rosemary essential oil was sufficient to achieve MIC values against *A. acidoterrestris* in the model medium. Although it was considered to have moderate inhibitory activity, the mixture of 15.62 µg/mL of rosemary oil and 1.95 ug/mL of nisin prevented the growth of bacteria and spore outgrowth for a 96 h incubation at elevated temperatures. The synergistic effect of nisin with the plant extract was also confirmed in research on *Piperaceae* C. Agardh plants, despite the fact that the extract alone effectively inhibited the growth of *A. acidoterrestris* in orange juice at a concentration of 15.62–62.50 µg/mL [109]. Similarly, the mixture of nisin and the hexane extract of *Matricaria chamomilla* L., when tested in vitro, was effective against a few strains of *Alicyclobacillus*, with minimum inhibitory concentration (MIC) and minimum bactericidal concentration (MBC) values in the range of 1.95–7.81 µg/mL, and a minimum sporicidal concentration (MSC) of 7.81 µg/mL. Even lower concentrations were effective in inhibiting *A. acidoterrestris* in orange juice [112]. An interesting study was undertaken by Alberice et al. [110] on the natural glycosidic compounds of *Sapindus saponaria* L. (*Sapindaceae*), which have proven antibacterial and antifungal properties. At concentrations of 400 and 500 mg/L, saponins were found to completely inactivate *A. acidoterrestris* after 72 and 24 h, respectively, when added to orange juice. The authors claimed that saponins could serve as a bactericide in the food industry; however, despite having no effect on the sensory characteristics of orange juice, their ability to cause foaming might be disadvantageous [110]. The potential of crude latex in the food industry was also suggested, as it inactivated five *A. acidoterrestris* strains at a concentration of 7.81 µg/mL, while MBC appeared to be strain-dependent [111].

Thymoquinone (TQ) exhibited a broad antimicrobial spectrum against vegetative cells, spores and biofilms formed on abiotic surfaces [41]. The minimum concentrations at which effective *Alicyclobacillus* growth inhibition was observed ranged from 32 to 64 μg/mL (MIC values). According to the time–kill assay, the addition of TQ at MIC and 2MIC (doubled MIC concentration) resulted in a bactericidal effect after 24 h, leading to cell morphology changes. The effect on spores was considered moderate, as even after a four-hour exposure, the number of spores decreased by approximately 1.2 CFU/mL at the highest concentration tested (16MIC—concentration 16 times higher than the MIC value). With regard to *Alicyclobacillus* biofilms formed on polystyrene and stainless steel surfaces, thymoquinone was reported to inactivate cells to a similar extent in a concentration-dependent manner [41].

High concentrations of citrus and neroli oils (>0.16% and >500 ppm, respectively) could also be effective against these acidophilic bacteria. However, due to their intense citrus aroma, which is not suitable for many food products, they would need to be added in small amounts, thereby reducing their antimicrobial activity [113]. A possible alternative is to use a combination of natural compounds and physicochemical methods to control the growth of *Alicyclobacillus* bacteria; however, the potential degradation of essential oils at high temperatures must be considered [40,113]. Research on essential oils and their components has shown that biologically active substances of natural origin can act as preservatives for fruit juices and pulps, ensuring their microbiological stability. Nevertheless, the phenomenon of bacterial adaptation to adverse external factors and the formation of bacterial biofilms make further research reasonable. The focus should be on verifying the antibiofilm capacity of substances of plant origin and their potential usage as agents to control bacterial presence in industrial environments.

## 4. *A. acidoterrestris* Biofilm

In both natural and industrial environments, microorganisms rarely occur as single cells, in a planktonic form. Instead, they usually colonize abiotic and biotic surfaces in clusters known as biofilms or biological membranes [114]. Biofilms can be structures that are either homogeneous (single-species) or heterogeneous (multi-species). Multi-species biofilms are characterized by a specific structure; individual niches and microenvironments are dominated by microorganisms depending on their nutritional requirements and adaptive abilities, as well as species interactions.

There are many factors that influence biofilm formation. One of these, particularly in industrial conditions, is fluid movement. Bacteria can adhere to surfaces of equipment when there is both static (laminar) and dynamic (turbulent) fluid flow [115]. In laminar flows, where shear forces are relatively low, three types of biofilm are observed: flat, columnar and mushroom-shaped [7,116]. In environments with strong shear forces (turbulent flow), biofilms are characterized by a smooth outer shell, an elongated ‘striped’ structure and tighter cell packing in the extracellular polymeric substance (EPS) matrix [115,116]. The degree and speed at which a surface is colonized by microbes largely depend on its structure. In food processing facilities, stainless steel is the material of choice; however, regular cleaning or disinfection with chemicals can cause corrosion, thereby increasing the surface’s porosity [117]. This allows microorganisms to colonize rough surfaces and areas that are difficult to access during sanitation processes [7]. Electropolished steel, i.e., steel that has undergone electrochemical treatment to reduce surface porosity and protect against corrosion, is less susceptible to microbial attack. Studies have shown that electropolished steel is colonized by bacteria much more slowly than conventional stainless steel [118].

Hydrophobic–hydrophilic interactions are an important factor in the formation of biological films. It is believed that microorganisms colonize hydrophobic surfaces (e.g., plastic) more quickly than hydrophilic surfaces (e.g., glass and stainless steel), and that their ability to adhere decreases as their hydrophobic properties decrease [8,119,120,121]. However, a clear relationship between microorganism adhesion and hydrophobicity is not always evident [122,123,124]. The composition of biofilms depends mainly on the qualitative and quantitative composition of the microorganisms introduced with the raw material, the availability of nutrients (including organic matter residues from semi-finished products), the effectiveness of sanitation processes, cross-contamination and environmental factors such as temperature, humidity and acidity [8,115,117]. Several studies have shown that the *Alicyclobacillus* genus (primarily *A. acidoterrestris*) can colonize technological surfaces such as stainless steel, PVC, glass and nylon [122,125,126].

*Alicyclobacillus* isolated from fruit juices and juice concentrates are usually introduced into processing plants with the raw material. Their heat-resistant endospores are activated by the pasteurization process, and the subsequent cooling of finished products to room temperature promotes spore germination and, consequently, cell multiplication. Some studies on *Alicyclobacillus* focus on determining their ability to form biofilms and their removal from production lines [122,125,126,127,128].

The first studies on the occurrence of *Alicyclobacillus* bacteria on technical surfaces were published in 2009 by Podolak et al. [127], who proved that *A. acidoterrestris* spores may be attached to stainless steel surfaces. Following the research by Orr and Beuchat [129] on the activity of disinfectants (200 ppm or 1000 ppm chlorine, 500 ppm acidified sodium chlorite, 2.0% or 4% hydrogen peroxide) against alicyclobacilli spores on the surface of apples, Podolak et al. [126] selected other chlorine-derived agents inhibiting germination of *A. acidoterrestris* spores on steel surfaces [126]. They observed a reduction in the number of spores by 1.3–2.6 log CFU/cm^2^ in the presence of a sodium hypochlorite solution (2000 ppm), a chlorine dioxide solution (200 ppm) and a commercial Vortexx solution (6.9% hydrogen peroxide, 4.4% peracetic acid and 3.3% caprylic acid; 2600 ppm) during short-term (1–2 min) contact between the steel surface and the disinfectants at temperatures of 40–90 °C. A study by Anjos et al. [125] confirmed the ability of *A. acidoterrestris* cells to adhere to abiotic surfaces, showing the colonization of stainless steel, PVC and nylon, finding stainless steel and nylon to be the most favorable surfaces for biofilm formation. Among the commercial disinfectants tested, quaternary ammonium salts demonstrated the most effective anti-sporicidal action, preventing spore germination at 82 ppm within 15 s contact, but peracetic acid expressed the highest effectiveness against *A. acidoterrestris* biofilm regardless of the surface tested, concentration or exposure time [125]. The work of Shemesh et al. [127] was groundbreaking—discovering that the ability of *A. acidoterrestris* strains to form biofilms was closely related to environmental acidity. As the pH decreased, decreases in cell motility and intense biofilm growth were observed. The substantial intensity of biofilm formation was observed at pH below 3.6. Ambient temperature was identified as another key factor in the colonization of surfaces by *A. acidoterrestris*. As temperature increased from 37 °C to 42 °C, changes in the pH ranges optimal for biofilm formation were observed from 3.0–3.5 to 3.0–3.5 and 5.0–5.5, respectively [127]. Research was also conducted on the adhesion phenomenon of environmental strains of *Alicyclobacillus* bacteria isolated in Poland [122]. According to the authors, alicyclobacilli are able to colonize glass surfaces and form mature biofilms after 48 h, the degree of which is more significant when dynamic culturing is performed. However, the correlation between biofilm formation and bacterial cell hydrophobicity was not obvious [122]. The phenomenon of colonization was subsequently proved for glass, polyvinyl chloride (PVC) and polystyrene surfaces, as well as the antibiofilm properties of clove, cinnamon and peppermint essential oils against *A. acidoterrestris* [4,130]. The level of *A. acidoterrestris* biofilm on the glass surface ranged from 2.48 log CFU/cm^2^ to 4.52 log CFU/cm^2^ and was about 1.0–1.5 log CFU/cm^2^ higher compared to the one formed on PVC within 72 h at 44 °C [130]. The clove essential oil was observed to be the most effective against *A. acidoterrestris* biofilm on technical surfaces (glass, PVC), causing its reduction by 25.1–65.0% in 0.05% (*v*/*v*) concentration [130]. The biofilm formation and eradication efficiency from polystyrene were dependent not only on the bacterial strain, type and concentration of essential oil but also on culture acidity and incubation temperature. In the polystyrene model, the strongest antibiofilm effect was noted for clove oil with the biofilm reduction ranging from 0.65 to 100% even at the lowest oil concentration, 0.5 MIC [4].

Moreover, the study by Dutra et al. [128] proved that *A. acidoterrestris* colonized both stainless steel and natural food-grade rubber surfaces at 28 °C within 48 h even at low initial cell loads. Additionally, in mature biofilm structures, spores were only formed following high initial loads of bacteria [128].

Due to the limited literature on the occurrence of biological membranes formed by alicyclobacilli in industrial environments, further studies on their ability to adhere to technical materials is needed, as is the search for effective, non-invasive methods of eradicating their biofilms in the food industry. An alternative method of combating bacterial biofilms in industrial environments is to use plant-derived substances. Unlike commercial disinfectants and chemicals, microorganisms do not develop resistance to essential oils or their components, which are a natural source of antimicrobial compounds.

## 5. Summary

*A. acidoterrestris* still remains a significant concern for the food industry, particularly in the production of fruit juice. The ability of the bacteria to withstand pasteurization and to colonize technological surfaces makes its control challenging. While numerous industrial practices can effectively reduce or eliminate *A. acidoterrestris* contamination, there is a growing interest in developing alternative, more natural control strategies that can replace or complement conventional chemical or mechanical treatments. Researchers continue to investigate combinations of standard and novel methods applied to eradicate microorganisms from industrial environments, simultaneously putting in effort to minimize sensory alterations in final products. Additionally, particular attention is being paid to exploring the antibacterial properties of natural compounds for inhibiting cell growth and preventing biofilm formation. Despite progress, substantial research is still required to fully understand and optimize these emerging approaches.

## Figures and Tables

**Table 1:** Natural active compounds tested for antimicrobial activity against *A. acidoterrestris*.

Factor	Active Component	Concentration	Medium	Reference
Chemical compound	Nisin	0.1–1.5 mg/L		[40]
	Limonene	0.52/3.7 mM	McIlvaine buffer	
	Citral	0.34/0.69 mM		
	Nisin + citral	0.3 mg/L + 0.34 mM 0.3 mg/L + 0.69 mM 1.5 mg/L + 0.69 mM		
	Cinnamaldehyde	10–500 ppm	acidified ME broth	[103]
	Eugenol			
	Limonene			
	Cinnamaldehyde	0–80 ppm	ME broth	[6]
		40 ppm	apple juice	
	Eugenol	0–160 ppm	ME broth	
		80 ppm	apple juice	
	Cinnamaldehyde + eugenol	20 ppm + 40 ppm	apple juice	
		20 ppm + 80 ppm		
Extracts	*Eucalyptus* spp. leaves	7.8–>250 mg/L	BAM broth	[104]
	Bicitro^®^	20–500 ppm	acidified ME broth	[105]
		80 ppm	apple juice	
	Neroli	20–500 ppm	acidified ME broth	
	Lemon	20–500 ppm	acidified ME broth	
		80 ppm	apple juice	
	Pomegranate	2.5–40 μg/mL	apple juice	[106]
	Grapeseed	0–3.6% *v*/*v*	apple juice	[107]
	Rosemary	1.0–125 μg/mL	apple juice	[108]
	Rosemary	1.95–1000 μg/mL	BAT broth	[39]
	Rosemary + nisin	3.90–4000 μg/mL + 1.95–125 μg/mL	orange juice	
	*Piper peltatum* *Piper marginatum*	0–62.50 μg/mL	orange juice	[109]
	*M. chamomilla* L.	0.0001–1000 μg/mL	BAT broth	[110]
		0.49–10,000 μg/mL	BAT broth, orange juice	
Other	Latex	0.49–1000 µg/mL	BAT broth	[111]
	Saponin	100–500 mg/L	orange juice	[110]
	Thymoquinone	8.0–512 μg/mL	AAM broth	[41]
Essential oil	Lemon oil	0.08–0.16% *v*/*v*	lemon juice concentrate, ME	[21]

ME—malt extract medium; BAM—Bacillus acidocaldarius medium; BAT—Bacillus acidoterrestris medium; AAM—Alicyclo acidoterrestris medium.

## Data Availability

No new data were created or analyzed in this study. Data sharing is not applicable to this article.
